# Intraoperative compensation of magnetic field distortions for fluoroscopic and electromagnetic hybrid navigation

**DOI:** 10.1007/s11548-022-02663-7

**Published:** 2022-05-23

**Authors:** Marco Cavaliere, Pádraig Cantillon-Murphy

**Affiliations:** 1 School of Engineering, University College Cork, College Road, Cork, Ireland; 2 Tyndall National Institute, Lee Maltings, Dyke Parade, Cork, Ireland

**Keywords:** Electromagnetic tracking, Fluoroscopy, Hybrid navigation, Calibration, Magnetic field modelling

## Abstract

**Purpose:**

Hybrid navigation is a promising technique which combines the benefits of optical or electromagnetic tracking (EMT) and fluoroscopy imaging. Unfortunately, the fluoroscopy system is a source of metallic distortion for the EMT system. In this work, we present a new method for intraoperative calibration and real-time compensation of dynamic field distortions. The method was tested in the presence of a fluoroscopy C-arm, and sub-millimetre errors were obtained after distortion correction.

**Methods:**

A hybrid navigation scenario was created by combining the open-source electromagnetic tracking system Anser EMT and a commercial fluoroscopy C-arm. The electromagnetic field generator was placed directly on top of the X-ray collimator, which introduced significant field distortion. Magnetic sensors were placed at known positions to capture the magnetic distortion, and virtual magnetic dipole sources were used to model the distortion magnetic field. The accuracy of the compensated EMT model was tested on a grid of test points.

**Results:**

Error reduction was demonstrated from 12.01 to 0.35 mm and from 25.03 to 0.49 mm, for horizontal and vertical sensor orientations, respectively, over a volume of 16 × 16 × 6 cm. It is proposed that such sub-millimetre tracking errors meet the needs of most endoscopic navigation tasks.

**Conclusions:**

We describe a method to model a magnetic field in real time, based on redundant electromagnetic field measurements, and we apply it to compensate for the distortion introduced by a fluoroscopy C-arm. The main limitation of the approach is the requirement for a high number of sensors, with possible occlusion of the operative space. Solutions might come from miniaturisation and wireless sensing.

## Purpose

Fluoroscopic X-ray imaging is currently considered the gold standard in minimally invasive vascular surgery, due to its high accuracy and reliability. However, the technology exposes the patient and the physician to ionising radiation.

Electromagnetic tracking (EMT) systems are a safe and cost-effective solution for the navigation of medical instruments, with no exposure to radiation and no requirements for a line of sight. Electromagnetic sensors available in the market can be as small as 0.3 mm in diameter [1], making it possible for their integration into medical devices used for minimally invasive surgery.

However, this technology has so far been limited by the lack of robustness against metallic materials, commonly present in the operating theatre. Magnetic interference introduces field distortions and consequent errors on instrument tracking.

A promising approach is to consider a hybrid navigation framework, where EMT is used for continuous navigation and X-ray is delegated to on-demand use during delicate manoeuvres or for confirmation. Unfortunately, the X-ray machine is a source of metallic distortion for the EMT system, the nature of which depends on the C-arm position and cannot be predetermined.

Recent works proposed online compensation approaches based on sensor arrays [2], simultaneous localization and tracking [3], artificial neural networks [4], and fusion of inertial and magnetic sensors [5].

In this work, we present a new technique for intraoperative calibration and real-time compensation of dynamic field distortions. The method was tested in a real distortion scenario, in the presence of a commercial fluoroscopy C-arm.

## Methods

A hybrid navigation scenario was investigated by combining the open-source electromagnetic tracking system Anser EMT [6] and the GE OEC Fluorostar 7900 (General Electric Company, Boston, Massachusetts, USA) fluoroscopy imaging system.

**Fig. 1:**
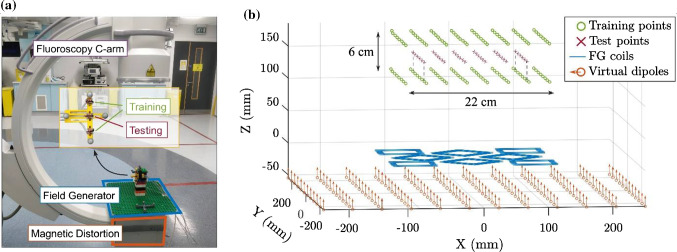
**a** Experimental set-up: the field generator (FG) was placed on top of the X-ray collimator, which introduced a large metallic distortion. Three three-axis sensors were used to measure the magnetic field on three parallel grids. **b** The top and bottom planes represent external sensors and were used to generate the field model which was then employed to find the position of the points in the midplane, for the EMT accuracy test with distortion compensation

The planar electromagnetic field generator was placed directly on top of the X-ray collimator, as shown in Fig. 1a. While this is not a realistic configuration, this set-up was chosen because it introduced the maximum field distortion and EMT error.

Three three-axis sensors (3DV11AOI-A-S0600J, Grupo Premo, Malaga, Spain) were moved on a predefined 8 × 8 grid, approximately 15 cm above the field generator, for a total of 192 measurement points distributed on three parallel planes. Magnetic measurements from the top and bottom planes were used to create a field model of the magnetic distortion, while the EMT accuracy was tested on the midplane, as shown in Fig. 1b.

Duplo blocks (The Lego Company, Billund, Denmark) were used to hold the sensors steadily during the acquisition time, but they did not provide reliable positional and rotational accuracy. Instead, optical tracking (Polaris Vega, Northern Digital Inc., Waterloo, Canada) was used as a position and orientation reference due to its high volumetric accuracy of 0.15 mm RMS [7]. The linear transformations between the sensors and the optical dynamic reference frame, and between the electromagnetic and optical systems’ coordinate frames, were obtained using a separate optically tracked stylus.

In the proposed technique, the calibrated field model is given by an array of virtual magnetic dipole sources, where the magnitude of each dipole is calculated based on the measured training data. For this experiment, the virtual sources have been virtually placed in proximity to the real physical position of the field generator and the metallic distorter under it, as shown in Fig. 1.

## Results

EMT accuracy results based on the central 6 × 6 test point array of the midplane are reported for the *Y* and *Z* oriented sensor coils, which are parallel and perpendicular to the field generator, respectively. The *X*-oriented coil gives very similar results to the *Y*-oriented case, and it is omitted for the sake of brevity.

**Fig. 2:**
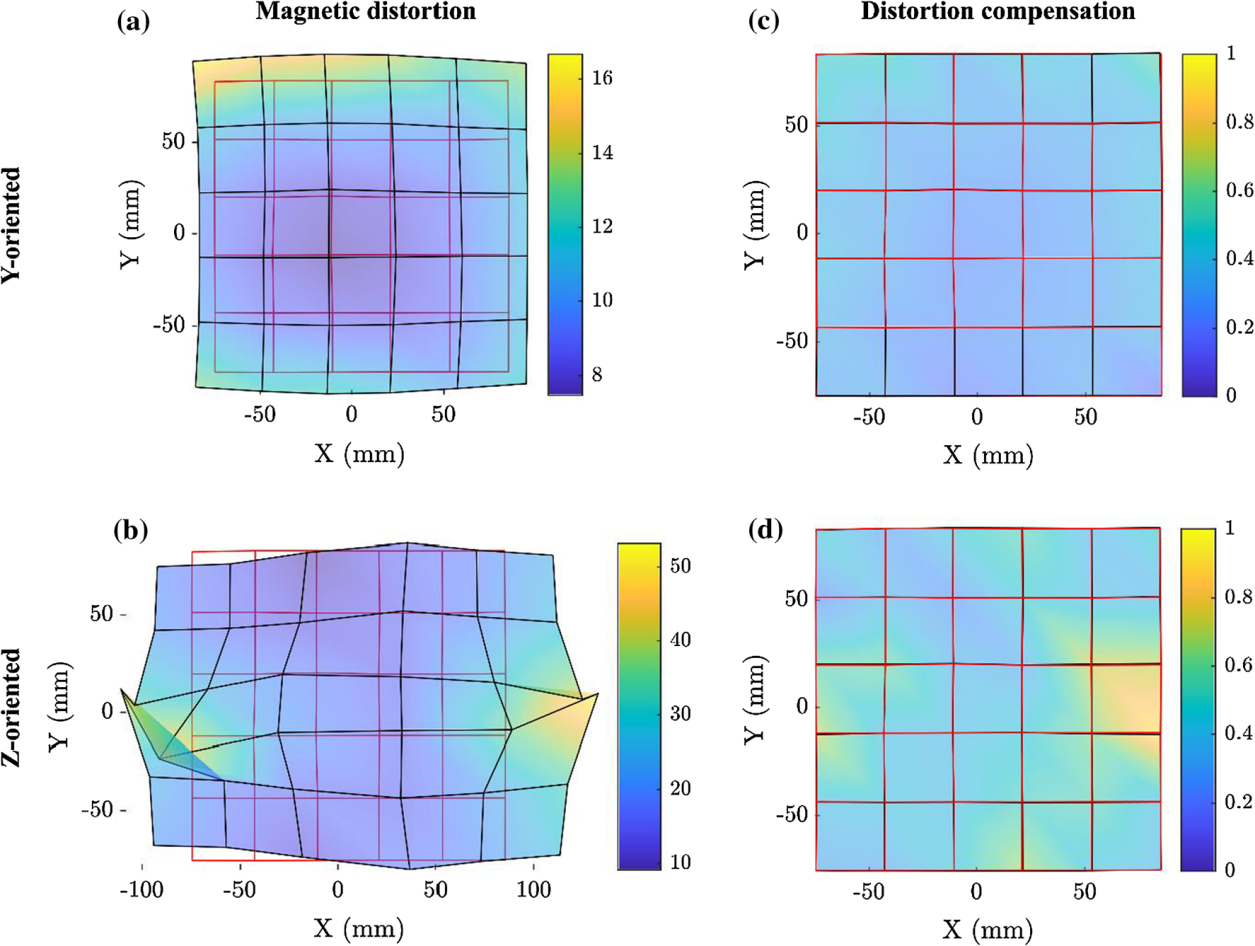
**a**, **b** EMT solution in the presence of the fluoroscopy system and **c**, **d** accuracy improvement obtained after calibration and correction, for a magnetic sensor oriented like **a**, **c** *Y* and **b**, **d** *Z* axis, respectively. The red grid is formed by joining the test points’ physical positions, the black grid represents the tracked points. The colour map (mm) also accounts for the error in the *Z* direction, which is not appreciable from the 2D plots

The effect of the C-arm distortion is visible in Fig. 2, where the grid of positions obtained from the EMT system (black) is compared to the real grid of test points (red). Figure 2 also shows the EMT solution when the distortion compensation technique presented in this work was applied.

The calibration algorithm took on average 20 ms to calculate the mutual inductance matrix between every virtual dipole and every training point, while it took 1.5 ms to find the optimal dipoles’ magnitudes, on a laptop with i5-11th Gen. Intel Core Processor. These values are compatible with real-time calibration because the data-driven magnetic field model is valid until the field distorters are moved. Moreover, the matrix can be calculated in advance if the positions of the training sensors do not change.

**Fig. 3:**
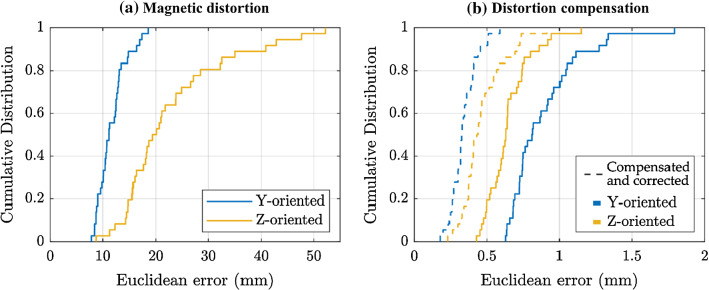
Empirical cumulative distribution function of the position error. **a** The high EMT errors are due to the presence of the fluoroscopy C-arm under the field generator. **b** Errors obtained when using the calibrated magnetic field model to solve the sensor position. The dashed lines show the further improvement obtained with the position correction based on the EMT solution of the points in the bottom plane

At each test point, the position error was calculated as the Euclidean distance between the electromagnetically tracked position and the reference position determined by optical methods. Figure 3 shows the empirical cumulative distribution function of the EMT error, comparing the distorted and the compensated cases.

A further correction was possible as the tracking errors of the points in the midplane (test set) are highly correlated with the errors obtained on the bottom plane, which are known because they are part of the training set. In other words, the training points of the bottom plane were electromagnetically localised, and the known displacement error was used to correct the EMT position of the test points. The results for the compensated and corrected tracking are shown in Fig. 3b.

To give an insight into the overall EMT position error within the tested region, some descriptive statistics are reported in Table 1: the root-mean-square error (RMSE), the mean error (ME), the median or 50th percentile (PRC50) and the 95th percentile (PRC95). RMS error decreased from 12.01 to 0.35 mm and from 25.03 to 0.49 mm, for horizontal and vertical sensor orientations, respectively.

**Table 1:** EMT position error

Orientation	Uncompensated		Distortion compensation		Compensated and corrected	
	*Y*	*Z*	*Y*	*Z*	*Y*	*Z*
RMSE (mm)	12.01	25.03	0.92	0.67	0.35	0.49
ME (mm)	11.71	22.87	0.89	0.65	0.34	0.47
PRC50 (mm)	11.15	19.71	0.81	0.63	0.33	0.43
PRC95 (mm)	17.22	46.21	1.33	0.94	0.51	0.73

Position error in the presence of the fluoroscopy system and accuracy improvement obtained with the calibrated magnetic field model and with the correction based on the bottom plane solution. The results are calculated from the 6 × 6 grid of test points on the midplane

While the required minimal accuracy should be evaluated based on the specific procedure, it is believed that sub-millimetre tracking errors meet the needs of most endoscopic navigation tasks [8].

## Conclusions

We described a method to compensate for electromagnetic distortions in real time, based on the creation of a new magnetic field model which accounts for the presence of external non-static metallic distorters. The magnetic field used for calibration is analytically modelled by virtual magnetic dipole sources, and it is trained to fit real magnetic measurements.

In particular, the technique can be applied to avoid the distortions introduced by a fluoroscopy system in clinical operations where fluoroscopy is used together with electromagnetic tracking. We showed that the presence of a C-arm unit can cause EMT errors as large as several centimetres, and we demonstrated that they can be reduced to less than 1 mm after calibration, in a volume of 16 × 16 × 6 cm.

The information required to perform the magnetic field calibration comes from a set of redundant sensors placed at known positions around the region of interest. In the envisioned application, the sensors will be integrated into external body-mounted patches which are localised using an undistorted tracking system, such as an optical camera. Intraoperative recalibration of their position can also be obtained from X-ray snapshots.

The main limitation of the current study is the close distance of 3 cm between the training and the test sets, whereas in a real setting these locations might not be accessible. A limitation of the method presented is the requirement for a high number of redundant sensors, with possible occlusion of the operative space. Solutions might come from an application-specific optimisation of the sensors’ positions, as well as miniaturisation and wireless sensing. Future work will include the design and development of the sensor patches and the preclinical validation of a hybrid navigation procedure.

## References

[1] ‘Aurora Sensors—NDI’. https://www.ndigital.com/products/aurora/aurora-sensors/. Accessed 22 Mar 2022

[2] Ramachandran B, Jain AK (2016) Distortion fingerprinting for EM tracking compensation, detection and error correction, US9522045B2, Dec 20, 2016. Accessed 21 May 2020. Available: https://patents.google.com/patent/US9522045B2/en

[3] Sadjadi H, Hashtrudi-Zaad K, Fichtinger G (2016). Simultaneous electromagnetic tracking and calibration for dynamic field distortion compensation. IEEE Trans Biomed Eng.

[4] Krumb H, Das D, Chadda R, Mukhopadhyay A (2021). CycleGAN for interpretable online EMT compensation. Int J CARS.

[5] Zhang Y, Wang K, Jiang J, Tan Q (2021). Research on intraoperative organ motion tracking method based on fusion of inertial and electromagnetic navigation. IEEE Access.

[6] Jaeger HA (2017). Anser EMT: the first open-source electromagnetic tracking platform for image-guided interventions. Int J Comput Assist Radiol Surg.

[7] ‘Polaris Vega—NDI’. https://www.ndigital.com/products/polaris-vega/. Accessed 30 Mar 2022

[8] Franz AM, Haidegger T, Birkfellner W, Cleary K, Peters TM, Maier-Hein L (2014). Electromagnetic tracking in medicine—a review of technology, validation, and applications. IEEE Trans Med Imaging.

